# A novel tablet-based application for assessment of manual dexterity and its components: a reliability and validity study in healthy subjects

**DOI:** 10.1186/s12984-022-01011-9

**Published:** 2022-03-24

**Authors:** Ayah Rabah, Quentin Le Boterff, Loïc Carment, Narjes Bendjemaa, Maxime Térémetz, Lucile Dupin, Macarena Cuenca, Jean-Louis Mas, Marie-Odile Krebs, Marc A. Maier, Påvel G. Lindberg

**Affiliations:** 1 Institut de Psychiatrie et Neurosciences de Paris, Inserm U1266, Université Paris Cité, 75014 Paris, France; 2 Centre de Recherche Clinique, GHU, GHU Paris Psychiatrie & Neurosciences, 75014 Paris, France; 3 Department of Neurology, GHU Paris Psychiatrie & Neurosciences, Université de Paris, Paris, France; 4 Evaluation Centre for Young Adults (Pépite), GHU Paris Psychiatrie & Neurosciences, Université de Paris, Paris, France; 5 Institut de Psychiatrie (Centre, National de la Recherche Scientifique [CNRS] 3557), Paris, France; 6 Université Paris Cité, INCC UMR 8002, CNRS, 75006 Paris, France

**Keywords:** Manual dexterity, Tablet application, Digital medicine, Measurement, Validity, Reliability, Inter-rater reliability, Aging

## Abstract

**Background:**

We developed five tablet-based tasks (applications) to measure multiple components of manual dexterity. Aim: to test reliability and validity of tablet-based dexterity measures in healthy participants.

**Methods:**

Tasks included: (1) Finger recognition to assess mental rotation capacity. The subject taps with the finger indicated on a virtual hand in three orientations (reaction time, correct trials). (2) Rhythm tapping to evaluate timing of finger movements performed with, and subsequently without, an auditory cue (inter-stimulus interval). (3) Multi-finger tapping to assess independent finger movements (reaction time, correct trials, unwanted finger movements). (4) Sequence tapping to assess production and memorization of visually cued finger sequences (successful taps). (5) Line-tracking to assess movement speed and accuracy while tracking an unpredictably moving line on the screen with the fingertip (duration, error). To study inter-rater reliability, 34 healthy subjects (mean age 35 years) performed the tablet tasks twice with two raters. Relative reliability (Intra-class correlation, ICC) and absolute reliability (Standard error of measurement, SEM) were established. Task validity was evaluated in 54 healthy subjects (mean age 49 years, range: 20–78 years) by correlating tablet measures with age, clinical dexterity assessments (time taken to pick-up objects in Box and Block Test, BBT and Moberg Pick Up Test, MPUT) and with measures obtained using a finger force-sensor device.

**Results:**

Most timing measures showed excellent reliability. Poor to excellent reliability was found for correct trials across tasks, and reliability was poor for unwanted movements. Inter-session learning occurred in some measures. Age correlated with slower and more variable reaction times in finger recognition, less correct trials in multi-finger tapping, and slower line-tracking. Reaction times correlated with those obtained using a finger force-sensor device. No significant correlations between tablet measures and BBT or MPUT were found. Inter-task correlation among tablet-derived measures was weak.

**Conclusions:**

Most tablet-based dexterity measures showed good-to-excellent reliability (ICC ≥ 0.60) except for unwanted movements during multi-finger tapping. Age-related decline in performance and association with finger force-sensor measures support validity of tablet measures. Tablet-based components of dexterity complement conventional clinical dexterity assessments. Future work is required to establish measurement properties in patients with neurological and psychiatric disorders.

**Supplementary Information:**

The online version contains supplementary material available at 10.1186/s12984-022-01011-9.

## Background

Manual dexterity can be defined as the ability to perform rapid, coordinated and precise finger movements, permitting grasping and manipulation of objects [1]. Dexterity is essential for many activities of daily living (ADL), including dressing, feeding, personal hygiene, and tool use, such as handling of a computer, tablet or mobile phone [2, 3]. Manual dexterity, exemplified by independence of finger movements [4], is therefore crucial for ADL and autonomy. Aging is associated with reduced dexterity [5–7] and with a negative impact on autonomy [8]. Finger and hand movements are impaired in a wide range of neurological, rheumatic and psychiatric illnesses [9–11] and associated with detrimental consequences on ADL [12, 13].

Hand function can be evaluated in many ways [14]. Conventional clinical measurements most often use functional measures (assess time and success rate) in grasping and manipulation of small objects, such as the Purdue Pegboard test [15], Box and Block test [16], Nine-Hole Peg Test [17, 18], Moberg Pick-up test [19] and Minnesota manual test [20]. These gross functional measures of dexterity contrast with neuroscientific investigations showing that dexterity is a multi-component construct that includes capacity to control force, to control timing of movements, to execute independent finger movements, and the ability to perform (and memorize) motor sequences. Several studies have previously investigated these key components of manual dexterity: (1) Control of force was evaluated for each finger [21] in precision [22] grip, for the hand in power grip [23, 24], as well as during grasp-and-lift tasks [25], (2) Finger independence, i.e., the capacity to move the fingers independently of each other, was assessed using kinematics [26, 27]. (3) Timing aspects were typically assessed by the capacity to synchronize finger movements to cues [28], and (4) Motor sequence performance was evaluated by execution of memorized or cued sequences of finger movements [29, 30]. Simultaneous quantification of such components in healthy subjects is rare and is lacking in conventional clinical (neurological) assessments of upper limb impairments [31]. Quantifying these complementary control variables, through a multi-component description of dexterity, allows for more accurate detection of impairments, such as in post-stroke motor status [32] and recovery [33], as well as in psychiatric [11] and neurodegenerative disorders [6]. However, these multi-component evaluations have so far required dedicated apparatus and software which can be challenging to incorporate in medical institutions.

A simpler and standardized tablet-based application for assessment of dexterity could be useful in several clinical settings, particularly when rapid screening is required. Previous multi-touch tablet-based tools have been developed to assess dexterity impairment [34–37]. Tablet tools may also be used to improve hand functioning during active rehabilitation [38–40]. Digital tools may provide readily accessible performance feedback with objective results, considered important for assessment and follow-up of dexterity impairments. While some previous digital tools have been shown to have good reliability and validity for detection of dexterity impairment [41, 42], other tablet-approaches investigated validity only [43]. Previous tablet-based dexterity assessments used specific types of hand and finger movements, for example only tapping [35] or simple tracing movements [36]. Others included a variety of functional tasks including drawing, tapping and tracing [42].

Based on our previous multi-component dexterity approach [32] we developed multiple tasks incorporated in a tablet-based application to quantify the different components of dexterity. In contrast to the existing and above cited tablet tools we focused on quantifying performance using five tasks, each one assessing a different component of dexterity. The five tasks included: (1) the finger recognition task to assess finger identification and action (effector) selection, (2) the rhythm task to assess timing of finger movements, (3) the multi-finger tapping task to assess the ability to perform independent finger movements, (4) the sequence task to assess the ability to perform and memorize a motor sequence involving multiple fingers, and (5) the line-tracking task (with/without a cognitive dual-task) to assess movement speed and accuracy (under different degrees of attentional load).

The aim of this study was to test the reliability and validity of these tablet-based dexterity measures in healthy participants. First, we determined inter-rater reliability of performance variables for each of the tablet tasks by comparing scores obtained from two separate test sessions. Second, to validate these performance measures we used three approaches: (1) assessing expected age-related decline in those measures, (2) comparing these measures to those obtained using a dedicated finger force-sensor device [32], and (3) correlating them to clinical assessments, including the box and block test (BBT) and the Moberg pick-up test. This was undertaken for a large array of measures extracted from the tablet tasks in order to select, via the criteria of validity and reliability, the most informative and appropriate performance measures for future use in clinical and nonclinical studies.

## Methods

### Study design

We investigated inter-rater reliability in one cohort of healthy subjects (N = 34) who performed the tabled tasks twice with two raters. Validity of task measures was studied in another cohort (N = 54) by correlating the tablet measures with age, clinical dexterity assessments (BBT and MPUT) and with measures obtained using a finger force-sensor device.

First, a repeated-measures design was employed to assess inter-rater reliability of tablet measures. To do so, data was collected on two separated test sessions by two different raters (rater 1 and rater 2). The minimal time interval between the two sessions was 2 h, the maximum was 19 days. For about one third of the subjects, the two measurements were obtained on the same day. Session circumstances and instructions were held constant.

Secondly, the use of multiple approaches was recommended for validation [44] and we studied validity of the tablet-based dexterity measures in three ways:

1. Through assessing age-related decline in tablet-based dexterity measures. Manual dexterity is known to decline with age [5–7]. Here we examined whether the tablet-based dexterity measures would detect this age-related decline.
2. By correlating tablet performance with that measured using a finger force-sensor device (Dextrain Manipulandum; https://www.dextrain.com). The Dextrain Manipulandum is a dedicated device to determine the key components of dexterity through various sensorimotor tasks [32].
3. By correlating tablet performance against scores of two conventional clinical assessments of dexterity: The Box and block test (BBT) of gross manual dexterity and the Moberg pick-up test of fine precision grip function. Both tests were performed in the same setting and same day as the tablet tasks. We expected weak relations between specific tablet-based measures and BBT and Moberg pick-up scores, since these latter two tests measure gross ability to pick-up and displace objects and have low variance in healthy adults.

### Participants

For testing of reliability, a total of 34 healthy subjects participated in this experiment. All recruited subjects were declared healthy, without neurological, orthopedic or other disorders affecting hand function. All participants provided informed consent and the study was approved by the ethical review board (CPP 2018-A01945-50). Demographic characteristics are shown in Table 1.

**Table 1:** Gender, age and handedness of participants and timing of experiments

	Inter-rater reliability experiment			Validity experiment		
	Male (N = 16)	Female (N = 18)	Total (N = 34)	Male (N = 24)	Female (N = 30)	Total (N = 54)
Mean age (years) ± SD [range]	34 ± 11 [5, 21–60]	36 ± 11 [5, 23–58]	35 ± 11 [5, 21–60]	50 ± 19 [20–78]	47.5 ± 17.5 [20–78]	49 ± 18 [20–78]
Handedness Right: Left: Ambidex	R14:L2:A0	R17:L1:A0	R31:L3:A0	R21:L1:A2	R27:L3:A0	R48:L4:A2
Mean interval between sessions (days) ± SD [range]	5.3 ± 6.2 [0–19]	3.9 ± 4 [0–11]	4.6 ± 5.1 [0–19]			

Validity of tablet-based measures was evaluated in a further 54 healthy subjects without neurological, orthopedic or other disorders affecting hand function. Participants provided informed consent and the study received ethical approval (CPP 2017-A01875-48). For studying age-related changes, the two samples were pooled (N = 88).

### Tablet-based dexterity tasks

**Fig. 1:**
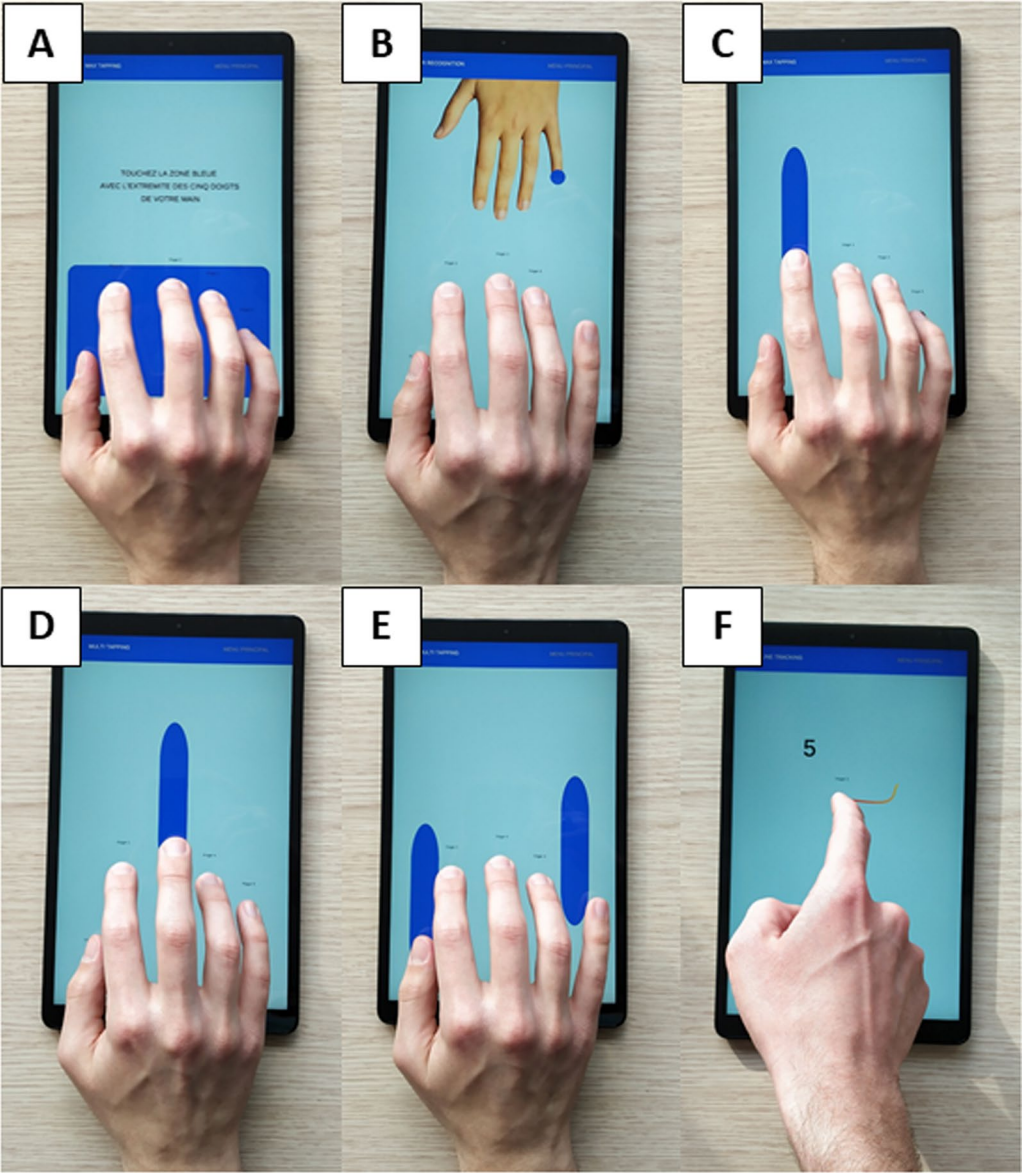
Illustration of the five tablet tasks. **A** Initial positioning of the hand and digits. Digits were positioned within the dark blue (working) zone for identification of each finger. Note: for the first four tasks the hand remained static during task execution, only the digits were supposed to move. **B** Finger recognition (FiRec) task. The dark blue circle on the avatar hand indicates the target finger with which to tap. The avatar hand is in the *mirror* condition, i.e., the left/right positioning of the digits of the avatar hand corresponds to the position of the subject’s digits. Two further avatar hand positions were used (*inverted* and *rotated*, not shown, see “Methods”) to introduce cognitive aspects (mental rotation) to finger identification and effector selection. **C** Rhythm tapping (RhyTap) task. A dark blue oval indicates the finger with which to perform the repetitive tapping according to regular auditory cues (or from memory). **D** and **E** Multi-finger tapping (MFTap). The subject is required to tap with one or two fingers (indicated by the dark blue oval) as fast as possible in reaction to this visual cue. **D** indicates a single-finger tap with the middle finger. **E** indicates a simultaneous two-finger tap with the thumb and little finger. **F** Line tracking (LT) task. The subject has to follow with his/her index finger a curved line segment (of 5 cm length) that moves smoothly but unpredictably from the lower part to the upper part of the screen and passing unpredictably from left-to-right and from right-to-left. The line progresses continually as the finger follows it (always with a 5 cm advance). The line tracking task under *dual-task* condition is shown with a number (#5) appearing during tracking. The subject is required to mentally subtract this number, and other numbers displayed later during the task, from 50 and provide the result at the end of the task

A standardized set of written and oral instructions was provided before each task (during both sessions and by each rater). Each session started with a brief introductory instruction, basic personal data collection, and familiarization with the tablet. At the beginning of each task the subject had to position his/her dominant hand within the dark blue rectangle of the tablet (Fig. 1A). Subjects were asked not to lift the hand off the screen until each task was completed. The position of each finger was detected within this rectangle for all five tasks. Specific instructions for each task were provided prior to each task. The order of the tasks was identical for all subjects (FiRec, RhyTap, MFTap, SeqTap and LineTr). Altogether, execution of the five tasks lasted about 20 min. The following five tablet (touch-screen) tasks were developed (Fig. 1):

1. *The Finger recognition task (FiRec). *Visual orientation of target cues has been shown to be useful in the study of internal representations and mental rotation of body position in disorders with impaired coordination [45]. This task was therefore devised to assess finger identification and action (effector) selection. After having positioned his/her hand within the working zone (Fig. 1A), the subject was instructed to perform a finger tap as fast as possible on the screen with the finger corresponding to the target finger indicated on the virtual, avatar hand (Fig. 1B). The avatar hand was oriented in three different ways that required different degrees of mental rotation: (1) *mirror-condition*: the avatar hand was positioned vertically and opposite the aligned hand of the subject (Fig. 1B), thus providing a spatial identity and entailed no mental rotation. (2) *inverted-condition*: the vertical avatar hand was left/right inverted (180° flip along the longitudinal hand axis) compared to subject’s hand, such that the target thumb was (left/right) aligned with the little finger of the subject’s hand. Correct effector identification and selection thus required a mental rotation. (3) *rotated condition*: the avatar hand was positioned horizontally (90° rotation) but the hand of the subject was (as usual) positioned vertically. Thus there was no simple left/right correspondence between the avatar hand and that of the subject. Correct task performance thus necessitated mental rotation. The Finger recognition task consisted of a total of 90 trials, 30 per condition. The order of conditions (mirror, inverted then rotated) was identical across all subjects and sessions. In left-handed subjects, the conditions were adapted so that the first condition was also mirrored.

*Analyzed variables*

• Reaction time (RT) capturing time taken for cognitive processing of the visual stimulus, decision making and execution of the motor response. Average RT (and SD) was calculated for each condition (*mirror*, *inverted* and *rotated*) as well as across the three conditions. We created additional variables to summarize changes in processing speed due to condition (orientation) of the avatar hand: RT cond.(*inverted*–*mirror*) and RT cond.(*rotated*–*mirror*).
• Average correct trials (N) indicating the number of correct taps across all 90 trials (range [0–1], 0 indicating no successful finger tap, 1 indicating complete success in all 90 trials).

2. *The Rhythm tapping task (RhyTap).* This task was designed to assess the timing of finger movements and involved adapting a previous finger force-sensor device task [32]. The subject was instructed to perform repetitive tapping movements on the screen (Fig. 1C) in synchrony with a regular auditory cue (at 1, 2, or 3 Hz). Each finger was tested separately, at all three frequencies (in the same order, 1, 2 and 3 Hz). The subject was required to tap for 10 s at the set frequency of the cue, (*cued* condition), and then to continue tapping for another 10 s at the same frequency without cue (*no_cue* condition).

*Analyzed variables*

• Intertap interval (ITI) for each trial and across all trials at each frequency (mean ITI and SD ITI, with or without cue).

3. *The Multi-finger tapping task (MFTap).* This task was developed to assess the ability to perform independent finger movements, again adapting a previously developed task [32]. The subject was instructed to perform a single-finger tap or a (simultaneous) two-finger tap, as fast as possible, in response to a visually cued target finger (colored oval shape indicating which finger, or which two fingers to tap simultaneously, Fig. 1D, E). A total of 90 trials were collected (30 single-finger trials, consisting of six trials for each finger, randomly mixed with 60 two-finger trials). For the ten possible two-finger combinations, 5 trials were recorded in each combination, for a total of 15 conditions (5 single finger and 10 two-finger combinations).

*Analyzed variables*

• Reaction time (RT) was computed for each correct trial and averaged across single and two-finger trials.
• Average correct trials (N) indicating the number of correct taps across all 90 trials (range [0–1], 0 indicating no successful finger tap, 1 indicating complete success in all 90 trials).
• Unwanted finger movements (N/trial) was quantified as the ratio of trials in which other finger/s than the target finger/s were actively moved across all trials. This measure described the degree of independence of finger movements.

4. *The Sequence tapping task (SeqTap).* This task was construed to assess the ability to perform and memorize a finger motor sequence and involved adapting a previous task [32]. A 5-tap sequence was displayed on the screen and the subject was instructed to tap, as fast as possible, to each cue with the respective target finger. Digit tap order was 3-4-2-5-1 (sequence A) or 4-3-5-2-1 (sequence B). The subject repeated the cued tapping sequence ten times (referred to as *acquisition* phase). Then the subject was required to tap this sequence from memory without any cue (referred to as *memory* phase). This was repeated five times.

*Analyzed variables*

• Successful trials across all trials (ST) and the number of successful taps within a trial (STT, range [0, 5]). The difference between the *acquisition* and the *memory* phase in ST and in STT indicated the degree of sequence memorization.

5. *The Line tracking task (LineTr)* Line-tracking is a sensorimotor task allowing measurement of upper limb motor speed and accuracy in experts [46] and in motor disorders [47]. We devised a line-tracking task on the tablet to assess movement speed and accuracy while following an unpredictably moving line on the screen with the index finger. This visuomotor task was repeated under a (cognitive) dual-task condition to evaluate the subject’s ability to control divided attention. The subject was instructed to perform line-tracking on the screen as fast and as accurately as possible (Fig. 1F). The lines to follow were randomly selected from a library of six random paths. During task execution, a randomly drawn pathway of a 5 cm long curved line appeared and moved on the screen. This segment would advance unpredictably, but only when the subject had his/her finger placed on and progressed with the line. Under the dual-task condition, visual distractors (geometrical forms) or integer numbers [0 to 9] appeared on the screen, while the subject was to continue tracking. Subjects were asked to ignore distractors, while subtracting the appearing numbers subsequently starting from 50. The task was performed three times: (1) condition *single-task* (simple tracking without dual-tasks). (2) as condition *dual-task (*mixing unpredictably distractors and numbers (to mentally subtract). (3) repeat of condition *dual-task*. Average duration, error and trade-off were computed for each condition and repetition.

*Analyzed variables*

• Time taken to complete the tracking (duration).
• Tracking error (distance in pixels from the midpoint of the target line to the position of the digit) for the entire path.
• Error/Duration was also assessed since we expected a speed-accuracy trade-off [48].

#### Force sensor dexterity testing using Dextrain device

The Dextrain Manipulandum (www.dextrain.com) was used as a separate and different procedure to quantify manual dexterity in the subjects. This device is equipped with a piston for each finger connected to a force sensor. The force applied by each finger on the corresponding piston was recorded using National Instruments acquisition card and tasks running on Labview (tasks same as in [32]). Only one of the original tasks was used, a visuo-motor multi-tapping task, to assess independence of finger movements in order to compare it to the tablet data obtained by the similar (but not identical) MFTap tablet task.

#### Clinical hand dexterity tests

The Box and Block Test [49] (BBT) was used to measure gross manual dexterity. The number of blocks displaced in one minute was counted.

The Moberg pick-up test was used to assess grip function. Time taken to place all 12 objects into the box was recorded [50].

#### Subjective ratings of difficulty and interest

We also investigated the reported comfort and interest after completion of all five tablet tasks. Subjects were asked to rate the comfort and interest of the tasks from 0 to 10 (from worst to best). This was obtained in 51 subjects.

#### Data analysis: pre-processing

**Fig. 2:**
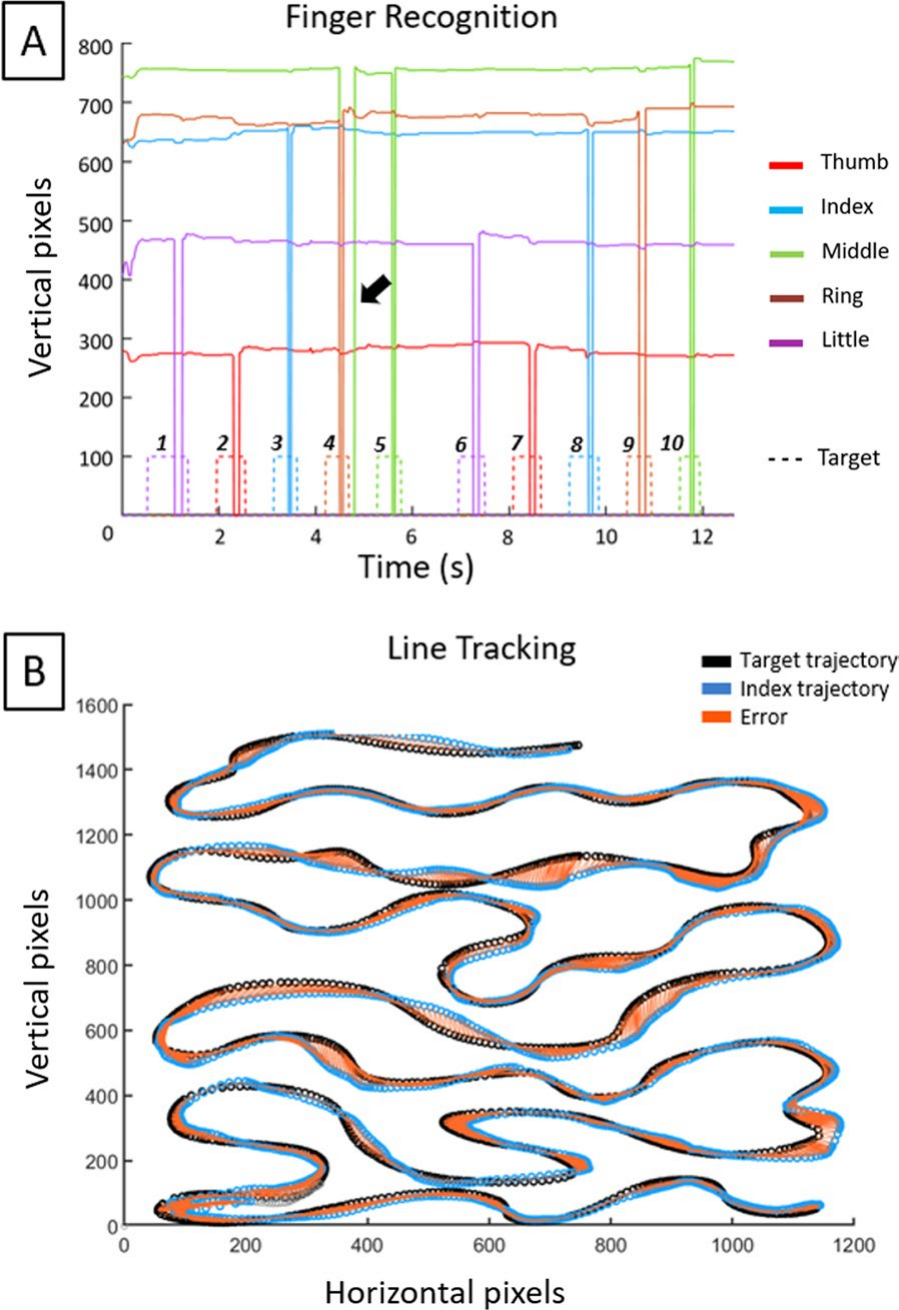
Raw data. **A** Finger recognition task. 10 successive trials. Stippled colored line: one color per target finger. Solid lines: one corresponding color per finger. Y-axis > 0: vertical position (pixel) of the respective fingertip in contact with the tablet (horizontal x-position not shown). Y-axis = 0: fingertip lift-off. A finger tap is indicated as a lift-off from the initial position on the tablet and a subsequent touch down. Correct tap: target and effector (finger) color correspond and tap occurs during target finger display. Note error tap on trial 4: target finger = ring finger, but the tap involved the ring and the middle finger. Similar type of data were recorded for Rhythm tapping and Multi-finger tapping. **B** Line tracking. Entire target trajectory (black trace) and effected index finger trajectory (blue) over the surface of the tablet. At any given time only a segment of 5 cm of the continuously but unpredictably moving target curve was visible. The tracking error (orange) corresponds to the area between the target and the effected trajectory

MATLAB (www.mathworks.com, v2019) was used for data analysis. Figure 2 shows example raw data for the Finger recognition (Fig. 2A) and Line tracking task (Fig. 2B). First, data were scrutinized for outliers:

*Finger Recognition task* data from two subjects were removed since they performed less than 50% of the total number of trials (i.e., < 45 trials per condition). Furthermore, trials with RT < 150 ms were considered anticipatory [51] and removed (this concerned 17 trials from four subjects out of a total of 23,760 trials).

*Multi-Finger Tapping task* data from two subjects were excluded since they responded in less than 50% of trials. Data from one more subject were excluded from the two-finger-tap condition due to responses in < 50% of trials. Again, single trial data with RT < 150 ms, or RT > 1 s were excluded. As a result, more than one hundred values were eliminated across the total of 7920 trials recorded across all subjects, i.e., < 2%.

Certain two-finger tap combinations were noticeably difficult to perform and resulted in missing data. When > 50% of these trials from a given two-finger combination were missing, data were eliminated. On complicated two-finger combinations we also remarked high variability between trials. In this case averages were calculated from the three fastest trials. Otherwise, the average was based on the five fastest two-finger combinations.

*Sequence Tapping task* visual data inspection revealed that some subjects did not follow the instructions, e.g. anticipated the memorized sequence prior to the starting cue, resulting in artefacts. This was corrected for by hand.

Finally, at the group level and after the above-mentioned pre-processing, multiple performance outliers with values > 1.5 times or < − 1.5 times the interquartile range (IQR) were excluded.

### Statistical analysis

*Reliability* Average ± SD were used as basic descriptive statistics for each raters’ data set. Normality of sampled data distribution was tested using Shapiro–Wilk test (p set to ≤ 0.05 for a significantly non-parametric distribution). 10 variables had a clear Gaussian distribution, 10 variables were identified as having Gaussian-like distribution with > 70% probability, and finally 9 variables did not show normal distribution. For these latter a logarithmic_Natural_ transformation was used to normalize them. However, some variables stayed non-normal even after transformation: these were analyzed with non-parametric statistical tests (e.g., Spearman correlation).

Both relative reliability, which indicates the maintenance of individual positioning in a given sample, and absolute reliability, which denotes the variation between measurements for each individual [53], were calculated. As recommended [54, 55], Intra-class correlation (ICC) was used to assess relative reliability. A one-way (rater) random-effect model ICC was applied as a measure of consistency between raters. Relative reliability scores, based on 95 CI, were classified as follows: ICC < 0.40 = poor, 0.40 > ICC < 0.59 = moderate, 0.60 ≥ ICC < 0.74 = good, and ICC ≥ 0.75 = excellent [56]. In addition to ICC_95,_ Pearson’s Correlation (r) or Spearman rank correlation (r_s_) were calculated.

Standard error of measurement (SEM) was calculated as an indicator of the absolute reliability (response stability). SEM was calculated as the SD of the difference of paired measurements divided by the square root number of trials [57].

$$SEM= SD/\surd n$$

Subsequently, SEM was used to calculate the minimal detectable change (MDC). MDC is clinical measure used as a cut-off value to differentiate a true change from a change due to measurement error [58]. MDC_95_ was calculated by multiplying the SEM with the z-score of the 95 CI (= 1.96) and the square root of 2 as follows:

$$MDC=1.96*SEM* \surd 2$$

**Fig. 3:**
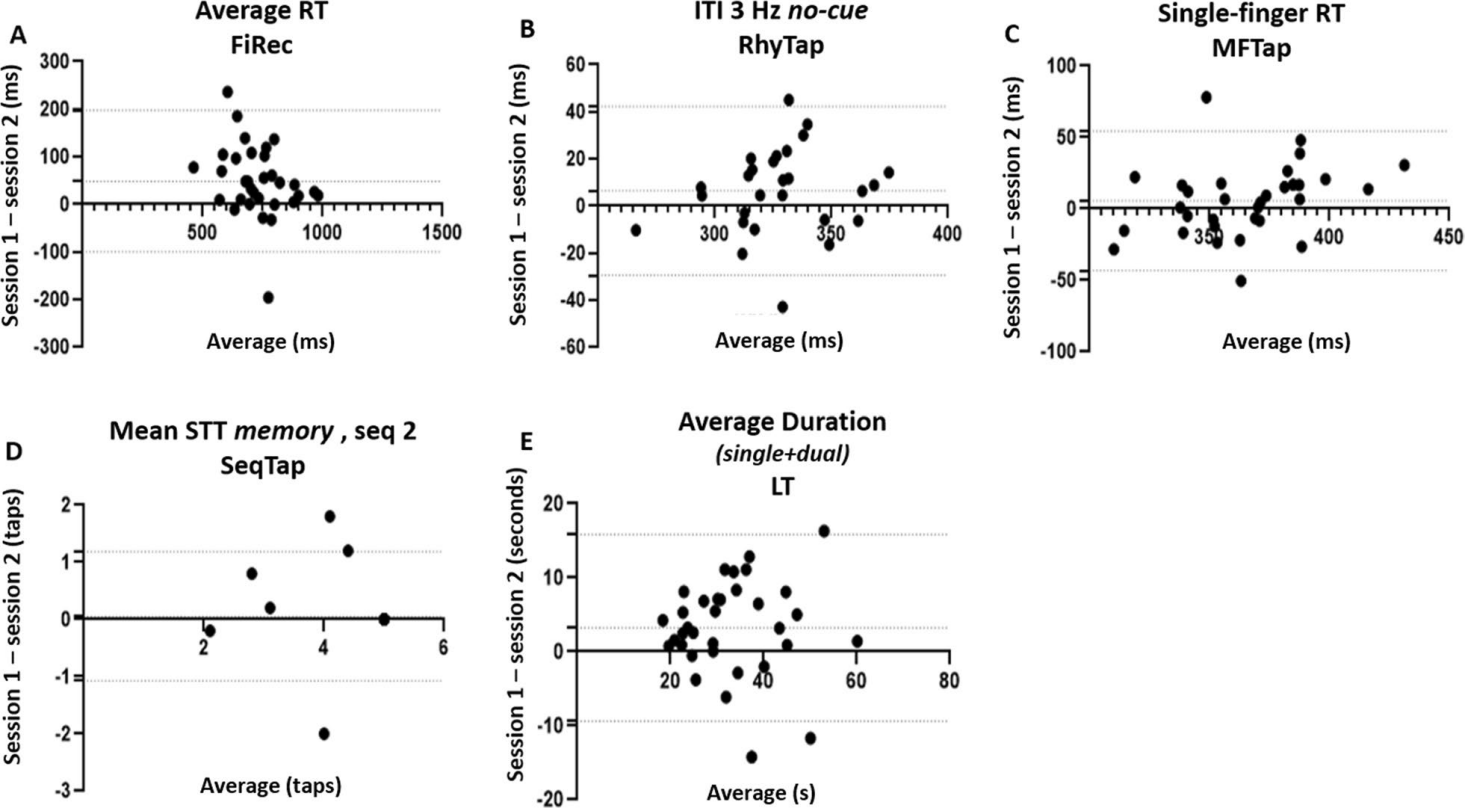
Bland–Altman plots (CI = 95) of the most reliable variable for each of the 5 tablet task. Change values are plotted against the same x-axis of the average of the two inter-rater sessions. **A** RT (average across the three different conditions of Finger recognition task, n = 32). **B** Intertap interval during 3 Hz rhythm task without auditory cues (N = 29). **C** RT during *single-finger* trials of the Multi-finger tapping task (N = 32). **D** Number of successful taps of second sequence during memorization (*memory*, n = 34). **E** Average duration of line tracking task (in s, N = 34). RT: reaction time in ms; ITI: intertap interval. Only Average Duration in Line tracking **E** showed a significant bias

Bland Atman plots with 95 CI were computed by plotting the paired difference between the two sessions against their mean (Fig. 3).

*Systemic bias* Systemic bias which reflects the degree of significant difference in performance that could be attributed either to fatigue or to learning needed to be studied. Paired *t-*test between the two sets of data was employed to confirm any significant difference indicating a systemic bias [59]. Alternatively, Wilcoxon paired test was used for not normally distributed data sets.

To evaluate the effects of task conditions on performance measures one-way ANOVAs were performed.

*Validation analysis* Spearman rank correlation was calculated for all variables in comparison with age (N = 88), BBT and Moberg test performance (N = 54). A p-value of 0.0016 was set to account for multiple comparisons (Bonferroni correction: 0.05/30). Relations between tablet-based dexterity measures and similar measures obtained with the Dextrain manipulandum were also determined using Spearman rank correlation.

*Inter-task correlation analysis* Spearman rank correlation between 12 selected (most reliable) variables from the 5 tablet-tasks was used to test for similarities (or specificity) in performance across tasks. Similarity (significant correlation) suggest presence of a common contributing factor to the two tested variables (components), leading to co-variation between performance variables. Absence of correlation suggests specificity of a task-variable not captured by the other task. The entire sample was used in this analysis (N = 88). A p-value of 0.0042 (0.05/12) was applied after Bonferroni correction.

## Results

All the tasks were completed by the subjects who reported a good level of comfort (mean = 7.6) and interest in the tasks (mean = 8.3) on a 0–10 scale.

### Relative and absolute reliability of tablet-based dexterity measures

*Finger recognition task* fastest reaction times occurred in the *mirror*-condition, followed by the *rotation* and the *inverted* condition. RT in the *mirror* and *rotated* conditions showed good relative reliability (RT *mirror*: ICC = 0.72; RT *rotated*: ICC = 0.71). RT had excellent reliability in *inverted* condition and for the average RT across all three conditions (RT *inverted*: ICC = 0.77; Average RT: ICC = 0.82). Both variability (SD of Average RT) and success rates (Average correct trial) showed moderate relative reliability (ICC = 0.58, 0.46, respectively). Reliability of change in reaction time as a function of hand orientation was good [RT cond. (*inverted-mirror*): ICC = 0.64; RT cond. (*rotated-mirror*): ICC = 0.62].

*Absolute reliability* the minimal SEM was observed in the RT *mirror* condition (SEM = 46.8 ms) and SEM was higher in the following two conditions (RT *inverted*: SEM = 84 ms; RT *rotated*: SEM = 73 ms). The change variables showed a similar range of measurement error (RT cond. *inverted-rotated*: SEM = 77 ms; RT cond. *rotated-mirror*: SEM = 69 ms). Average correct trials showed very small SEM of only 2/100 trials.

*Rhythm tapping task* The inter-tap interval during the cued phase (ITI *cued*) showed increasing relative reliability from 1 to 2 Hz and to 3 Hz (ICC = 0.31, 0.66, 0.72, respectively). Reliability was similar at 2 Hz and 3 Hz when performing without cues and slightly improved at 1 Hz (ITI 1 Hz.*no_cue*: ICC = 0.55; ITI 2 Hz.*no_cue*: ICC = 0.45; ITI 3 Hz.*no_cue*: ICC = 0.76).

Absolute reliability showed lowest SEM values at 3 Hz (ITI 3 Hz.*cued*: SEM = 11.5 ms; ITI 3 Hz.*no_cue*: SEM = 13 ms).

*Multi-finger tapping task* The reaction time in both *single* and *two-finger* combinations showed good reliability (RT *single-finger*: ICC = 0.68; RT *two-finger* combination: ICC = 0.60). Degree of unwanted fingers movements showed poor reliability in both *single* (ICC = 0.19) and *two-finger* combinations (ICC = 0.09).

Absolute reliability was best for *single finger* RT (SEM = 18 ms) compared to *two-finger* combinations (SEM = 29 ms).

*Sequence tapping task* successful replication of finger tapping sequences showed good reliability during the memorization phase and when comparing performance between acquisition and memorization. This held for the 1st sequence (ST.*acquisition.*seq1: ICC = 0.72; STT. (*acquisition-memory.*seq1): ICC = 0.69) and for the 2nd sequence (ST.*memory.*seq2: ICC = 0.76; ST.(*acquisition-memory.*seq2): ICC = 0.66).

*Absolute reliability* the overall range of SEM was about half a successful tap (out of 5 repetitions/25 expected successful taps) in both sequences of the memorization phase (ST.*memory*.seq1: SEM = 0.57; ST.*memory*.seq1: SEM = 0.41).

*Line tracking task* Excellent relative reliability was found for time taken to complete the task in *single-task* line-tracking and in *dual-task* line-tracking (duration *single*: ICC = 0.78; duration *dual*: ICC = 0.76) and in average duration (ICC = 0.84). However, change between single and dual-task Line tracking showed poor reliability (duration *dual-single*: ICC = 0.34). Average error and Error/Duration showed good reliability (Table 2).

**Table 2:** Reliability of 35 performance measures extracted from the five tablet tasks

Variables	N	Rater1 (Mean ± SD)	Rater2 (Mean ± SD)	ICC (95CI)	Corr. (r) or (rs)	SEM (95%CI)	MDC (95%CI)	p-value
Finger recognition								
1. RT *mirror* cond. (ms)	**32**	635 ± 85.7	606.9 ± 88.16	0.72	0.71***	46.79	129.7	0.20
2. RT *inverted* cond. (ms)		857.4 ± 160.6	794 ± 181.3	0.77	0.77***	83.86	232.44	0.14
3. RT *rotated* cond. (ms)		771.9 ± 124.2	716.7 ± 162.6	0.71	0.59***	72.93	202.15	0.01^†^
4. **Average RT** (ms)		754.8 ± 110.7	705.9 ± 132.9	0.82	0.82***	53.6	148.55	0.12
5. RT cond. *inverted-mirror* (ms)		222.4 ± 127	187.2 ± 124	0.64	0.56***	76.85	213.01	0.08
6. RT cond. *rotated-mirror* (ms)		136.9 ± 86.5	109.8 ± 129.5	0.62	0.63***	68.86	190.6	0.10
7. **SD of Average RT** (ms)		200 ± 58.1	179.4 ± 52.9	0.58	0.50**	33.81	93.71	0.02^†^
8. **Average Correct Trial** (N)		0.97 ± 0.03	0.98 ± 0.03	0.46	0.40*	0.02	0.57	0.01^†^
Rhythm tapping								
9. ITI 1 Hz *cued* (ms)	**27**	924.8 ± 40.3	944.7 ± 25.7	0.31	0.22	28.30	78.44	0.01
10. ITI 2 Hz *cued* (ms)		476.2 ± 25.5	484.5 ± 21.4	0.66	0.66***	13.9	38.7	0.01
11. **ITI 3 Hz** *cued* (ms)		287.1 ± 19.7	311.6 ± 23.1	0.72	0.55**	11.59	32.13	< 0.0001
12. ITI 1 Hz *no_cue* (ms)		969.2 ± 81.3	975.9 ± 69.3	0.55	0.54**	51.47	142.71	0.63
13. ITI 2 Hz *no_cue* (ms)		505.1 ± 28.3	504.2 ± 29.4	0.45	0.44*	21.69	60.11	0.88
14. ITI 3 Hz *no_cue* (ms)		330.9 ± 26.8	324.5 ± 24.3	0.76	0.75***	12.95	35.89	0.08
15. **SD ITI 3 Hz** *no_cue* (ms)		4.31 ± 1.94	3.35 ± 1.31	− 0.02	− 0.02	1.67	4.63	0.045
Multi-finger tapping								
16. **RT** *single-finger* (ms)	**32**	367.5 ± 33.8	361.9 ± 26.6	0.68	0.68***	17.64	48.90	0.21
17. **RT** *two-finger-combin.* (ms)	**31**	410.9 ± 54.3	399.5 ± 37.9	0.60	0.64***	28.90	80.11	0.18^†^
18. RT (*two-single*) (ms)		447.8 ± 33.8	384.5 ± 24.7	0.44	0.45*	22.42	62.14	0.28
19. Unwanted movements *single-finger* (N/trial)		0.06 ± 0.08	0.06 ± 0.07	0.19	0.18	0.07	0.19	0.93
20. Unwanted movements *two-finger combination* (N/trial)		0.54 ± 0.19	0.62 ± 0.22	0.09	− 0.09	0.2	0.55	0.17
21. **Correct trials** *single-finger* (N/trial)		0.98 ± 0.04	0.94 ± 0.18	− 0.05	− 0.06	0.13	0.36	0.91
22. **Correct trials** *two-finger combination* (N/trial)		0.90 ± 0.12	0.94 ± 0.07	− 0.09	− 0.11	0.1	0.28	0.09
Sequence tapping								
23. STT.*memory*.seq1 (N correct trials)	**34**	4.38 ± 1.2	4.6 ± 0.9	0.72	0.48**	0.57	1.61	0.31
24. STT.*acquisition-memory*.seq1 (N)		0.5 ± 1.2	0.36 ± 0.84	0.69	0.29	0.59	1.62	0.65
25. STT.*memory*.seq2 (N)	**29**	4.7 ± 0.78	4.64 ± 0.84	0.76	0.65***	0.41	1.14	0.59
26. STT.*acquisition-memory*.seq2 (N)		0.24 ± 0.77	0.27 ± 0.74	0.66	0.64***	0.45	1.26	0.38
27. **Mean STT. ***memory*. seq1 + 2 (N)		4.47 ± 0.95	4.54 ± 0.85	0.84	0.84	0.45	1.26	0.47
Line tracking								
28. Duration *single-task* (s)	**34**	36.6 ± 14	32.4 ± 14.8	0.78	0.72***	6.77	18.77	0.01^†^
29. Duration *dual-task* (s)		33.7 ± 10.1	30.9 ± 9.2	0.76	0.75***	4.85	13.44	0.02
30. **Average Duration** *single* + *dual* (s)		34.7	31.4 ± 10.7	0.84	0.84***	0.76	2.11	0.002^†^
31. Duration (*dual-task*-*single-task*) (s)		− 2.9 ± 8.4	− 1.5 ± 8.2	0.34	0.28	0.34	0.94	0.51
32. Average Error (N pixels)		81.6 ± 23.6	72.3 ± 17.9	0.60	0.57***	12.51	34.68	0.002^†^
33. Error/duration *single-task* (N/s)		2.6 ± 1.2	2.4 ± 1	0.69	0.62***	0.69	1.91	0.32^†^
34. Error/duration *dual-task* (N/s)		2.4 ± 1.1	2.4 ± 1.1	0.69	0.62***	0.59	1.63	0.44^†^
35. Average Error/duration (N/s)		2.5 ± 1.1	2.4 ± 1.1	0.76	0.64***	0.54	1.50	0.87^†^

Variables in **bold**: selected for inter-task correlations (Additional file 1: Table S1). Terms in *italics* denote various task conditions. N = sample size, SD: standard deviation, ICC: Intraclass correlation, RT: reaction time (ms), ITI: intertap interval, *cued*: with auditory cues, *no_cue*: without auditory cues. STT: number of successful tap trials. Corr: correlation coefficient between rater1 and rater2 values, r: Pearson’s correlation, rs: Spearman’s correlation. Note: asterisks indicate level of significance: *p ≤ 0.05, **p ≤ 0.01, ***p ≤ 0.001. p-values followed by † are based on non-parametric Wilcoxon paired test, otherwise paired t-test. ICCs based on not log-transformed data showed only small deviations from the corresponding log-transformed data, suggesting good robustness (N = 10 variables, mean absolute difference: r = 0.04, range = [0.0, 0.06])

Comparable absolute reliability was found in single and dual-task line-tracking duration (Duration *single*: SEM = 6.7 s; duration *dual*: SEM = 4.8 s). However, the SEM was < 1 s when averaged across conditions (Duration Average: SEM = 0.76 s). Average task error showed a SEM of 12.5 pixels.

#### Systemic Bias in tasks

Systematic bias is reported to inform on learning effects between the two sessions. Significant learning was found in few variables (Table 2). In the Finger recognition task an enhanced average success rate across all conditions was found in session 2 (Wilcoxon paired test, Average correct trial; p = 0.008), as well as faster responses (RT *rotated* cond.3; p = 0.005; p < 0.01) and lower variability of response time (SD of Average RT, p < 0.02).

In the Rhythm tapping task, the *cued* inter-tap interval increased significantly at second test for the three different rhythm frequencies (1 Hz and 2 Hz: p < 0.01, 3 Hz: p < 0.0001). However, no change was found in tapping frequency in the *no_cue* phase.

No statistically significant changes were observed in Sequence tapping or Multi-finger tapping variables.

Finally, in the Line-tracking task both the duration and error showed significant reductions at second testing, indicating faster and more accurate line-tracking performance in session 2 (Wilcoxon paired test, p = 0.002; Table 2). The trade-off variable, Error/Duration, did not show any changes across session.

The degree of agreement between measures obtained in session 1 and session 2 is illustrated in Bland–Altman plots (Fig. 3) for one variable per task: the one with the highest relative and absolute reliability. Only Average Duration in Line tracking showed significant bias (Fig. 3E).

#### Minimal detectable change (MDC) in tasks

MDC values were obtained for all tablet performance measures indicating the amount of change required to surpass the expected measurement error (Table 2).

### Validity of tablet-based dexterity measures

Validity was investigated by three approaches: (1) by verifying whether the tablet-based performance measures would capture known age-related decline in dexterity, (2) by comparing them to quantitative measures obtained with an alternative device (Dextrain) quantifying independence of finger movements, and (3) by correlating the measures with clinical assessments of dexterity.

*Age effects* multiple performance measures showed significant correlations with age. The strongest correlations were obtained in the Finger recognition, Multi-finger tapping and Line-tracking tasks (Table 3). In the Finger recognition task, average reaction time and variability (SD) of responses increased significantly with age (p < 0.0001). Age explained about 16% of the variance in reaction time and 25% of the variation in variability (SD of RT). In the Multi-finger tapping task the number of correct *two-finger* taps decreased significantly with age (p < 0.001). The number of unwanted finger movements in *single-finger* taps tended to increase with age (p < 0.01). In the Line-tracking task, both duration and Error/Duration increased significantly with age (p < 0.0001). No significant relation to age was found in the Rhythm task and the Sequence Tapping task.

**Table 3:** Validity of 30 performance measures extracted from the five tablet tasks

Variable	Correlation with age (r_s_, N = 88)	Correlation with BBT (r_s_, N = 54)	Correlation with Moberg test (r_s_, N = 54)
Finger recognition			
1. RT *mirror*	0.37*	− 0.18	0.37
2. RT *inverted*	0.40*	− 0.40	0.25
3. RT *rotated*	0.41*	− 0.11	0.06
4. Average RT	0.43*	− 0.32	0.22
5. RT cond. (*inverted-mirror*)	0.17	− 0.35	0.06
6. RT cond. (*rotated-mirror*)	0.23	− 0.03	− 0.10
7. SD of average RT	0.51*	− 0.11	0.24
8. Average Correct trial	− 0.33	0.03	− 0.05
Rhythm tapping			
9. ITI 1 Hz. *cued*	0.10	− 0.01	− 0.03
10. ITI 2 Hz. *cued*	0.27	− 0.28	0.25
11. ITI 3 Hz. *cued*	0.24	− 0.34	0.26
12. ITI 1 Hz. *no_cue*	0.01	0.14	− 0.07
13. ITI 2 Hz. *no_cue*	0.25	− 0.12	0.10
14. ITI 3 Hz. *no_cue*	0.12	− 0.11	0.09
Multi-finger tapping			
15. RT *single-finger*	0.23	− 0.27	0.27
16. RT *two-finger*	− 0.10	− 0.32	0.07
17. RT (*two-single finger*)	− 0.26	− 0.08	− 0.05
18. Unwanted movements *single-finger*	0.28	− 0.11	− 0.12
19. Unwanted movements *two-finger*	− 0.13	0.24	− 0.04
20. Correct trial *two-finger*	-0.394*	0.24	− 0.04
Sequence tapping			
21. STT. *acquisition*	-0.24	0.01	0.24
22. STT. (*acquisition-memory*)	0.08	0.04	-0.33
Line tracking			
23. Duration *single-task*	0.55*	− 0.14	0.1
24. Duration dual-task	0.51*	0.09	0.03
25. Average Duration	0.55*	0.02	0.10
26. Duration cond. (dual-single task)	− 0.15	0.13	− 0.21
27. Average Error	− 0.05	0.13	− 0.11
28. Error/duration *single-task*	− 0.46*	0.02	− 0.12
29. Error/duration *dual-task*	− 0.38*	− 0.06	− 0.01
30. Average Error/duration	− 0.53*	0.11	− 0.18

N = sample size, SD: standard deviation, RT: reaction time, ITI: intertap interval, *cued*: with auditory cues, *no_cue*: without auditory cues. STT: (number of) successful tap trials. (r_s_) Spearman’s correlation, BBT: Box and block test, Moberg: Moberg pick-up test right hand. *significant correlation with p ≤ 0.00166 (Bonferroni corrected p < 0.05). Terms in *italics* denote various task conditions

**Fig. 4:**
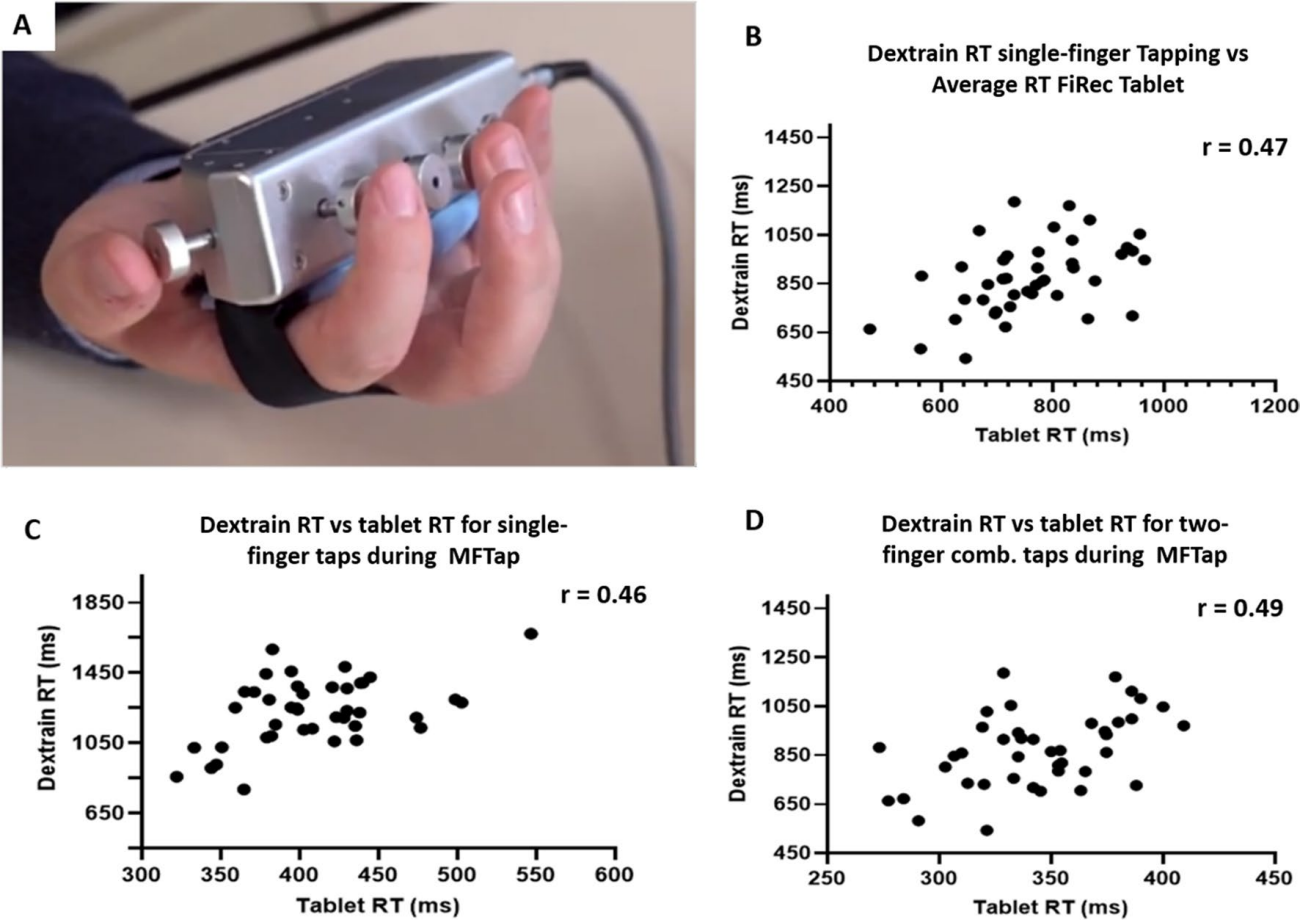
Validity: relation between Tablet and Dextrain task variables quantifying dexterity. **A** Dextrain device with one piston (force sensor) per finger. **B** Correlation between RT during single-finger tapping using Dextrain and the average RT in the tablet finger recognition task (FiRec): N = 43; r = 0.47, p = 0.002. **C** Correlation between Dextrain RT and tablet RT for *two-finger taps* during multi-finger tapping (MFTap): N = 41; r = 0.46, p = 0.002. **D** Correlation between Dextrain RT and tablet RT for *single-finger taps* during multi-finger tapping: N = 41; r = 0.49, p = 0.001. N: number of subjects, r: Pearson’s correlation coefficient

*Comparison to measures obtained using the Dextrain manipulandum* (Fig. 4A) a significant positive correlation of average reaction time between the tablet-based Finger recognition task and the single-finger taps using the Dextrain manipulandum (multi-finger tapping task, r = 0.47, p = 0.002, Fig. 4B) was found. Reaction times in the Multi-finger tapping tasks, on the tablet and with Dextrain, correlated significantly for both single (r = 0.46, p = 0.002, Fig. 4C) and dual-finger taps (r = 0.49, p = 0.001, Fig. 4D).

*Comparison to clinical measures* no or weak correlations were found between tablet measures and BBT-score or Moberg pick-up test score (Table 3).

### Inter-task correlations between performance variables

Whether and to what extent the different tablet-based performance measures represent different non-redundant components of dexterity was investigated through inter-task correlations. These were computed for a total of 12 variables (bold in Table 2), i.e., for the most reliable variable of each task, and for seven other selected key variables (Additional file 1: Table S1).

Only a minority (15) out of the 66 computed correlations were significant. Of those 15, 7 correlations concerned variables within the same tablet task (Additional file 1: Table S1, within rectangles). Thus, the remaining 8 significant correlations were observed between different task measures (Additional file 1: Table S1, outside rectangles). Finger recognition task: Average Reaction time correlated with single-finger reaction time in Multi-finger tapping task (r = 0.41, p < 0.0001). And the success rate (average # of correct trials) correlated positively with success rate during Multi-finger tapping, for single-finger taps (r = 0.33, p = 0.003) and for two-finger taps (r = 0.40, p < 0.0001). Variability (SD) of RT in Finger recognition correlated negatively with success rate in multi-finger tapping, for single-finger taps (r = -0.67, p < 0.0001) and two-finger taps (r = -0.69, p < 0.0001), and showed moderate correlations with RT in Multi-finger tapping (*two finger*, r = -0.30, p = 0.01) and with average duration in Line Tracking (r = 0.35, p < 0.001. Rhythm tapping: the variability (SD at 3 Hz *no_cue*) correlated moderately with RT in Multi-finger tapping (*two finger*, r = 0.38, p = 0.002). No other significant inter-task correlations were observed (Additional file 1: Table S1).

## Discussion

This study was undertaken within the framework of developing a digital tablet-based tool for quantifying manual dexterity. The study was designed to investigate reliability and validity of multiple tablet-based behavioral measures of manual dexterity in healthy subjects. Dexterity was conceived as a multi-dimensional concept whose components need operationalization. Reliability was determined through an inter-rater design and (content) validity trough a comparative approach using a variety of methods [44] including (1) detection of age-related changes, (2) comparison to measures from a previously used alternative technology to assess dexterity components [6], and (3) comparison to clinical scores. This was undertaken for a large array of measures extracted from the tablet tasks in order to select the most informative and appropriate performance measures for future use in clinical and nonclinical studies.

Generally, good to excellent relative inter-rater reliability was found for about 70% of the extracted tablet variables (ICC > 0.6). In three of the five tasks and in about 40% of the variables a significant correlation with age was observed. A good relation to kinetic (Dextrain device) measures was obtained in comparable variables. As expected, the relation to clinical dexterity scores (BBT, Moberg-test) was weak. Together, the findings provide first evidence supporting the validity of the tablet dexterity measures. We discuss some methodological considerations, advantages and limitations of the tablet approach for the measurement of dexterity components.

### The novel tablet tasks for measurement of manual dexterity

We used five complementary tablet tasks in order to capture and quantify key components of dexterity. A multitude of performance variables (> 30) was extracted to quantify these components with the rationale to select the most appropriate among those according to criteria of reliability and validity. We also computed inter-task correlations between these variables to establish potential redundancy among them. Twelve variables across the five tasks were found to capture relevant quantitative aspects of dexterity without being redundant, or only partially so (Additional file 1: Table S1). Clearly, variables extracted from Rhythm and Sequence tapping, relying on predictable selection and timing of finger taps (effector), did not correlate with those from the other three tasks where the cue was unpredictable (Finger recognition, Line tracking, and Multi-finger tapping). Furthermore, tasks involving cognitive resources other than sensorimotor, such as memory-based rhythm or sequence tapping, provided measures largely uncorrelated to the other three cognitively less demanding tasks. However, reaction time measures across the different tasks tended to correlate among each other indicating robust measurement of psychomotor processing.

Together, this suggests that the evaluated components of dexterity are largely independent of each other and complementary, and that they together provide a multi-component, rather than a unidimensional functional/clinical description of dexterity, typified by a single score. That each task provides at least one non-redundant measure indicates that the five tasks are complimentary for quantifying dexterity at this fine-grained, multi-component level. Nonetheless, we do not claim that they capture necessarily all aspects of dexterous control: extracting elements of force control [32] would likely lead to an even more comprehensive description, with potentially higher sensibility, while kinematic approaches can provide information on grasp strategies [60, 61] not captured by the tablet.

### Consistency of measurements: inter-rater reliability

Generally, good to excellent relative inter-rater reliability (ICC > 0.6) was found for the majority of the extracted tablet performance variables. Average reaction times during Finger recognition showed high ICC values (ICC = 0.82) similar to ICC values reported using other digital devices for clinics (ICC = 0.84) [67]. The few exceptions concerned tasks requiring control of independent finger movements, i.e., Multi-finger tapping and Sequence tapping. In both tasks, the amount of correct trials increased during the second session. This might be related to task difficulty: on the one hand, a simple task tends to give rise to ceiling effects [62], on the other hand, difficult tasks tend to provoke floor effects. Indeed, in cued sequence tapping more than 50% of the subjects performed at the top score for number of correct trials. And Multi-finger tapping showed a floor effect, with only 35% of the scores indicating presence of unwanted finger movements, as well as a ceiling effect with 25% of the subjects with top scores in number of correct trials. In the future, these two tasks may be optimized by adjusting their difficulty.

Systemic bias may account for latent learning effect in test–retest paradigms [59]. Two tasks, Multi-finger tapping and Sequence tapping, showed no learning, i.e., stable performance across sessions. However, reaction time and speed measures were more susceptible to systemic bias. There was significantly faster performance in the second session of the cued Rhythm tapping task, with more accurate intertap intervals. Speed also increased in the second session of the Line tracking task. A few other variables were also affected, such as the number of correct trials in Finger recognition and decreased line tracking error. A more extensive familiarization may further improve test–retest reliability and reduce systemic bias [63] for these tasks.

### Validation of tablet-based measures

Validity was probed with several complementary approaches, as recommended [44]. First, by whether we could obtain an age-related decline in performance with the tablet measures, as previously shown more generally [51, 64] and with other measures of dexterity [5, 6]. Indeed, three tablet-tasks (Finger recognition, Multi-finger tapping and Line Tracking) revealed the expected performance decrease with increasing age in several variables, and in particular in reaction time, suggesting validity with respect to these age-related changes. However, no such age-effect was observed in Sequence tapping and Rhythm tapping. Motor sequences have been shown to be affected by age, though these studies used longer (more difficult) sequences [45]. In line with our results, rhythmic (cue free) tapping has been shown to vary little with age [46].

Second, the tablet measures were related to comparable measures obtained with the Dextrain device. This device was previously used to quantify impairments of manual dexterity in stroke patients [32], in patients with schizophrenia [11], and in the elderly [6]. Measures that were comparable, i.e., those extracted from the Finger recognition and the Multi-finger tapping task relating to effector (action) selection and independent finger movements [65], indicated moderate [66] validity (r > 0.45).

Third, correlations with clinical tests of dexterity (BBT and the Moberg pick-up test) were evaluated. There is currently no consensus gold standard for assessing dexterity in disease [31]. We expected weak correlation here since BBT and Moberg pick-up tests have been developed to differentiate the degree of dexterity between healthy and pathological subjects with hand and finger movement impairments. Reliability and validity of these clinical tests resides in the clinical domain [16, 49, 52]. Furthermore, the BBT is a gross measure of dexterity not requiring fine dexterous manual skills (no obligation for using precision grip, no object handling other than grasp required, and dependence on proximal arm movements). Indeed, no significant correlations were found between the tablet performance and those two scores. Had we included patients with impaired dexterity then we would likely have found a relation to the Moberg-test, which requires manual dexterity, such as fine control of (precision grip) force and precise finger movements, as well as a moderate correlation with the more gross BBT score. We have previously shown that force-based measures of dexterity in stroke patients were well correlated with the Moberg pick-up test score [32]. Another factor may be that the Moberg (and the BBT) capture an overall global level of dexterity, incorporating multiple aspects of dexterous control. This would suggest that a global average measure of dexterity, across the various tablet tasks, would capture a more similar global score of dexterity. Future work is needed to design such a global dexterity score, particularly when comparing to neurological or psychiatric patients. With this in mind, the tablet and the Moberg test might better be considered two complementary approaches capturing different aspects of dexterity.

### Study limitations

Some limitations of this study need be considered: a first limitation concerns the various time intervals between test and retest, ranging from 2 h to 19 days. This may have introduced additional variability not accounted for. Second, the familiarization process (mentioned above) may not have been sufficient. It relied on written and oral instructions, but could have been prolonged and better illustrated using videos playing on the tablet. Third, the tablet measures do not (currently) incorporate force measures and force measures might have further improved the correlation between tablet measures and the Dextrain measures. These problems, as well as potential task and ergonomic optimization, will be taken into consideration for future development of the tablet application. Finally, we did not find a correlation between tablet dexterity measures and the scores of clinical scales (BBT and Moberg test). However, this most likely reflects a genuine difference between assessing dexterity components vs. more gross functional clinical scores of manual dexterity. More detailed clinical dexterity tests, for example the Southampton Hand Assessment Procedure [68], may have provided more similar results to those obtained with the tablet and future studies on this are indicated.

### Clinical implications

A future aim is to quantify impairments of dexterity in neurological and psychiatric patients using the tablet. Particularly relevant in the clinical domain are the issues of sensitivity and of minimal detectable change (MDC). The present data suggest that the tablet-based quantification has a better sensitivity than current clinical scales (e.g., detection of age-related decline). While the here obtained MDC values in healthy subjects indicate performance changes beyond the expected measurement error, determining MDC values in patients aims at detecting the minimal change that makes a functional difference in the lives of patients. We expect higher MDC values in patient groups since they typically show higher variability in performance. Tablet-based evaluation of dexterity in disease seems to be feasible, at least in stroke [37].

## Conclusion

Tablet-based measures of manual dexterity extracted from five complementary tasks showed, except for independent finger movements, good-to-excellent inter-rater reliability (ICC ≥ 0.60). A majority of these measures showed age-related decline and correlations to respective measures obtained via a dedicated finger force-sensor manipulandum supporting adequate validity of the various tablet measures. Furthermore, these different components of dexterity were not detected by conventional clinical dexterity assessments, such as the BBT and Moberg-test, suggesting higher sensibility for the tablet measures. Subsequent investigations are required to establish measurement properties in patients with neurological and psychiatric disorders.

## Supplementary Information

**Additional file 1.** Inter-task correlations.

## Data Availability

The datasets used and/or analyzed during the current study are available from the corresponding author on reasonable request.

## Abbreviations

ADL: Activities of daily living
BBT: Box and block test
CI: Confidence interval
FiRec: Finger recognition task
Hz: Hertz
ICC: Intra-class correlation
ITI: Inter-tap interval
LineTr: Line tracking task
MDC: Minimal detectable change
MFTap: Multi-finger tapping task
MPUT: Moberg pick up test
RhyTap: Rhythm tapping task
RT: Reaction time
SD: Standard deviation
SEM: Standard error of measurement
SeqTap: Sequence tapping task
ST: Successful trials
STT: Successful taps within a trial
