# Supplementary material for: A novel tablet-based application for assessment of manual dexterity and its components: a reliability and validity study in healthy subjects
Source: J Neuroeng Rehabil. 2022 Mar 24;19:35. doi: 10.1186/s12984-022-01011-9 (PMC8953393; doi:10.1186/s12984-022-01011-9)
Supplement: Supplementary file 1 — Additional file 1. Inter-task correlations. [file 12984_2022_1011_MOESM1_ESM.docx]

**Additional materials**

**Table S1.** Inter-task correlation*.* Correlation among 12 performance variables selected from the 5 tablet tasks (Table 2): Finger recognition, Rhythm tapping, Multi-finger tapping, Sequence tapping (SeqTap), Line tracking.

|  |  | **Finger recognition** | | | **Rhythm tapping** | | **Multi-finger tapping** | | | | **Seq. tapping** | **Line tracking** | |
| --- | --- | --- | --- | --- | --- | --- | --- | --- | --- | --- | --- | --- | --- |
|  |  | **Mean RT** | **Mean # correct trials** | **SD of mean RT** | **ITI 3Hz *no_cue*** | **SD of ITI 3Hz *no_cue*** | **RT *single finger*** | **RT *two finger comb.*** | **Correct # trials *single finger*** | **Correct # trials *two finger*** | **Mean STT *memory*** | **Mean duration** | **Mean error** |
| **Finger recognition** | **Mean RT h** | • |  |  |  |  |  |  |  |  |  |  |  |
|  | **Mean # correct trials** | ***-0.43**** | • |  |  |  |  |  |  |  |  |  |  |
|  | **SD of mean RT** | 0.55* | ***-0.46**** | • |  |  |  |  |  |  |  |  |  |
| **Rhythm tapping** | **ITI 3Hz *no_cue*** | 0.08 | -0.02 | 0.00 | • |  |  |  |  |  |  |  |  |
|  | **SD of ITI 3Hz *no_cue*** | 0.12 | -0.06 | -0.09 | 0.04 | • |  |  |  |  |  |  |  |
| **Multi-finger tapping** | **RT *single finger*** | ***0.41**** | 0.06 | -0.05 | 0.08 | 0.15 | • |  |  |  |  |  |  |
|  | **RT *two finger*** | 0.27 | 0.26 | ***-0.30**** | 0.12 | ***0.38**** | ***0.46**** | • |  |  |  |  |  |
|  | **Correct # trials *single fi.*** | -0.05 | ***0.33**** | ***-0.67**** | 0.08 | 0.16 | 0.30 | ***0.42**** | • |  |  |  |  |
|  | **Correct # trials *two fi.*** | -0.12 | ***0.40**** | ***-0.69**** | 0.13 | 0.15 | 0.15 | ***0.43**** | ***0.74**** | • |  |  |  |
| **Seq. tap.** | **Mean STT *memory*** | -0.06 | 0.25 | -0.22 | 0.10 | 0.06 | -0.01 | 0.07 | 0.12 | 0.20 | • |  |  |
| **Line tracking** | **Mean duration k** | 0.22 | -0.26 | ***0.35**** | 0.18 | -0.10 | 0.10 | -0.06 | 0.21 | -0.21 | -0.21 | • |  |
|  | **Mean error n** | -0.15 | 0.10 | 0.03 | -0.03 | 0.25 | 0.01 | -0.01 | -0.11 | -0.02 | -0.02 | -0.05 | • |

RT: reaction time, ITI: intertap interval, *cued*: with auditory cues, *no_cue*: without auditory cues. STT: (number of) successful tap trials. Values in the matrix represent Spearman correlations (r_s_). ***Bold italics****: significant correlation at p≤0.0042 (Bonferroni corrected p<0.05). Correlations within a thick-bordered rectangle represent within-task correlations, those outside represent inter-task correlations.
